# Plasma mineral profiles and hormonal activities of normal cycling and repeat breeding crossbred cows: A comparative study

**DOI:** 10.14202/vetworld.2015.42-45

**Published:** 2015-01-13

**Authors:** Abhijit Barui, Subhasis Batabyal, Sarbaswarup Ghosh, Debjani Saha, Saibal Chattopadhyay

**Affiliations:** 1 Department of Veterinary Biochemistry, Faculty of Veterinary and Animal Science, West Bengal University of Animal and Fishery Sciences, Kolkata, West Bengal, India; 2 Sasya Shyamala Krishi Vigyan Kendra, Ramakrishna Mission Vivekananda University, Narendrapur, Kolkata, West Bengal, India

**Keywords:** crossbred cow, hormone, mineral profile, normal cyclic, repeat breeding

## Abstract

**Aim:**

The present study was carried out to compare the associated role of micro minerals and hormones in repeat breeding animals with the normal crossbred cows.

**Materials and Methods:**

Blood samples were collected from 10 normal cycling and 10 repeat breeding crossbred cows of Ramakrishna Mission Ashram, Narendrapur to study the plasma mineral profile and hormonal activities.

**Results:**

Zn was found to be highly significant (p<0.01) between the two groups. Follicle stimulating hormone (FSH) and progesterone showed significant (p<0.05) difference in repeat breeding animal from the normal cyclic animal, whereas no significant differences were observed in Ca, P, Cu, Se, Co, luteinizing hormone and estradiol level.

**Conclusion:**

It may conclude that repeat breeding condition of crossbred cows in farm condition is mainly due to the low level of progesterone, FSH and zinc.

## Introduction

India is in possession of about 199 million cattle of which 33.06 million was estimated as crossbred [1]. These animals play an important role in the Indian livestock economy. In spite of the huge cattle population in our country, the performance of milk production is far below compared with the same in other developed countries, Reproductive problems are common cause of profit loss to the farmers as far as dairy farming and as well as beef production. The success of the dairy cattle economy lies in proper and optimal reproductive rhythm of each individual cow and buffalo in the herd within normal physiological range [2]. Longer dry period and reduced calving and lactations during the life span of an animal causes heavy economic loss. Infertile animal mean a direct loss in milk production, whereas reduced calf crops hamper the selection efficiency in long-term dairy herd improvement [3].

The breeding efficiency of dairy cows is lowered by a number of reproductive disorders like endometritis, anestrus and repeat breeding resulting in great economic losses to the dairy farmers [4]. Repeat breeding is one of the major problems affecting the reproductive efficiency. The cause of repeat breeding is multifaceted; most common causes are genetical, anatomical defects of the reproductive tracts, hormonal imbalances, infections such as clinical/subclinical endometritis and poor management [5]. A multifactorial problem involving a number of extrinsic factors, as well as intrinsic factors coupled to the individual animal, could also be a cause. Since several factors affect the incidence of repeat breeding in dairy cows, it is difficult to make generalizations regarding predominant causes.

Normal levels of various biochemical constituents are indispensable for normal function of various systems of the body including the reproductive system. Changes in these constituents have been blamed for reproductive failures. No study has yet been conducted on increasing repeat breeding problems in dairy herds of South 24 Parganas district of West Bengal, which is characterized by varying degree of mineral-deficient soils [6].

Therefore the present study was undertaken to investigate the plasma profile of certain minerals and hormones in cycling and repeat breeding cows, so as to compare and define the probable biochemical etiological factors involved in the infertility problem of the farm managed crossbred cow of this area.

## Materials and Methods

### Ethical approval

The study was approved by the University Animal Ethics Committee constituted for research purpose.

### Animals

A total of twenty adult clinically free of disease crossbred (Jersey × Sahiwal) cows were randomly selected for the present study. The cows were maintained at dairy farm of Ramakrishna Mission Ashram, Narendrapur, Kolkata. These experimental animals were kept under uniform feeding and managerial condition, and fed with ad libitum green grass/fodder with free access to water. The cows were categorized into two groups: The first group bred and conceived normally after no more than three inseminations. The second group was those animals that did not conceive after three or more inseminations, but had clear vaginal mucous discharge at estrus. The study was approved by the University Ethics Committee constituted for research purpose.

### Sample collection

Blood was collected in sterilized vacutainer with anticoagulant from the jugular vein of each animal. The plasma was separated by centrifugation (1500 ×*g* for 20 min) and collected in a sterile vial and preserved at −20°C until analysis. The blood samples from the cows were collected only after 12 h of the onset of estrous.

### Estimation

Ca, P were determined spectrophotometrically (SYSTRONICS-119) according to the methods described by Bagainski [7], Fiske and Subbarow [8] respectively. Concentrations of Cu, Zn, Se and Co were estimated in all plasma samples using atomic absorption spectrophotometer (VARIAN AA 240) by the method as described by Sandel [9] and Arenza *et al*. [10]. Plasma progesterone, follicle stimulating hormone (FSH), luteinizing hormone (LH) and estradiol concentrations were estimated by ELISA kit (Monobind, Lilac) as per the standard protocol provided with the kit. The trial was replicated thrice. Each replication of the trial was conducted in 3 weeks interval.

### Statistical analysis

The experimental data were analyzed using Student’s t-test [11] for determining the significance of the changes from the controls at 5% and 1% levels. The analysis was performed using SPSS software (version 10.0), SPSS South Asia Pvt. Ltd.

## Results and Discussion

Results of the experiment have been presented in Table-1. Among the hormones, plasma FSH was significantly low in the repeat breeder cows from the normal cyclic cows. Kaswan and Bedwal [12] reported that the zinc may lead to reduced GnRH secretion by hypothalamus resulting decrease of LH and FSH. The low-level estradiol and progesterone in the repeat breeding animal supports the present findings where FSH and LH have got their role to maintain a low level of ovarian estradiol and progesterone. In this study plasma, LH and estradiol hormone level were found non-significantly (p>0.05) between normal cyclic and repeat breeder cow. The findings were corroborated with Saleh *et al*. [13]. The non-significant lower level of estradiol in repeat breeding animal than the normal cyclic animal in the present study may be due to the low level of cholesterol that affects the steroidogenesis in the ovaries. Significant (p<0.05) variations of plasma progesterone level were observed between normal cyclic and repeat breeder cow. Result was in agreement with Akhtar *et al*. [14]. Ahmed *et al*. [15] also reported that serum progesterone level was significantly (p<0.05) low during the mid-luteal phase of the estrous cycle in repeat breeder buffaloes compared with normal animals. On contrary, Kumar *et al*. [16] revealed that the serum progesterone was significantly higher in sub-estrus cows than in the anoestrous and repeat breeders and even normal cyclic cows. Progesterone is a key hormone for investigating reproductive activity, and it is usually applied to monitor ovarian function in farm animals, since it reflects the different development stages of corpora lutea after ovulation. In luteal cells prostaglandin F2α mobilizes intracellular calcium, generates reactive oxygen species, depletes ascorbic acid levels, inhibits steroidogenesis and ultimately induce cell death [17]. As a result the corpus luteam regresses and the progesterone secretion decreases, which may be the cause the significantly lower value in repeat breeding animal than the normal cyclic animal.

**Table-1:** Plasma biochemical values in normal cyclical and repeat breeding cows (mean±SE).

Parameters	Normal cyclic cow	Repeat breeder cow
FSH (mIU/ml)	1.8906±0.2421	1.1688±0.2003**
LH (mIU/ml)	0.3644±0.054	0.3190±0.041*
Estradiol (pg/ml)	54.5278±3.8407	44.9000±3.7104*
Progesterone (ng/ml)	5.6111±0.7472	3.3600±0.4988 **
Calcium (mmol/L)	1.8986±0.073	1.8895±0.0718*
Phosphorus (mmol/L)	2.0861±0.1035	1.9278±0.1006*
Copper (µmol/L)	30.5578±1.5889	30.7660±1.3135*
Zinc (µmol/L)	20.4300±0.4450	16.8860±0.3418***
Cobalt (µmol/L)	66.6478±1.8457	66.3920±3.0315*
Selenium (µmol/L)	0.7630±0.1135	0.6414±0.0671*

* Non-significant P>0.05,

** Significant P<0.05,

*** Highly significant P<0.01, SE=Standard error, FSH=Follicle stimulating hormone, LH=Luteinizing hormone

Non-significant (p>0.05) variations of plasma calcium level were observed between cyclic and repeat breeder cow. Akhtar *et al*. 2014 [18] pointed out significantly higher calcium concentrations in normal cyclic buffaloes than the repeat breeders. Concentration of Ca in this study was in accordance with Kalita and Sarmah [19], Ramakrishna [20] who found Ca to be non-significant between the two groups. Calcium appears to affect reproduction indirectly in animals. Among the major minerals, Ca is known to influence the animal’s ability to use other trace elements. Its influence on certain enzyme systems may be mediated via disruption of reproductive efficiency. Non-significant (p>0.05) variations of plasma phosphorus level were observed between normal cyclic and repeat breeder cow. This finding was not in agreement with Kalita and Sarmah [19], Kumar *et al*. [21] and Chaurasia *et al*. [22]. A non-significant difference was reported by Yadav *et al*. [23] which corroborated with this study. Phosphorus is essential for transfer of biological energy, particularly through adenosine triphosphate, and deficiency of it may arrest the phenomenon of fertilization, and, this in turn, may cause early embryonic death resulting in the repeat breeder and anestrus conditions of animals [21]. Non-significant (p>0.05) variations of plasma copper level were observed between cyclic and repeat breeder cow. The present findings were in agreement with Haedoo *et al*. [24]. In contrary, Akhtar *et al*. [25], Modi *et al*. [26], Akhtar *et al*. [18] reported that copper was highly significant in normal cyclic animal than the repeat breeder animal. Highly significant (p<0.01) variations of plasma zinc level were observed between cyclic and repeat breeder cow. Accordingly Das *et al*. [27] the concentration of Zn varied significantly (p<0.01) among the animals with normal ovulation, anovulation and delayed ovulation. Narnaware and Sirothia [28] found serum zinc values to have no significant difference between acyclic and normal cyclic animals. In contrary Kumar *et al*. [15] observed significantly higher zinc content in anoestrus and repeat breeders than the subestrus and normal cyclic cows. Zn plays a key role in maintaining the integrity of the epithelia of the reproductive organs, which is necessary for embryo implantation [29]. Non-significant (p>0.05) variations of plasma cobalt level were observed between cyclic and repeat breeder cow. A non-significant difference was also found by Haedoo *et al*. [24] between cyclic and non-cyclic Surti buffaloes. Hidiroglou [30] reported that the cobalt is required to ensure fertility in ruminant. Low cobalt levels reduce the storage of copper in the liver resulting in interference with the activity of zinc, iodine, and manganese. In this study, non-significant (p>0.05) variations of plasma selenium level were observed between the two groups. The present findings were not in accordance with Ahmed *et al*. [15]. There was significantly lower serum selenium levels in anoestrus buffaloes (p<0.05) was reported by Akhtar *et al*. [25]. Hidiroglou [30] reported that in cattle and sheep, selenium deficiency is associated with the reduced fertility.

## Conclusion

The results presented above can provide supportive evidence about the importance of hormones and minerals in the resumption of repeat breeding condition. The results demonstrated that the incidence of repeat breeding case of crossbred cows in the farm condition was associated with a significantly low level of some hormones like FSH and progesterone. Among the minerals, zinc was found to play the most vital role to cause repeat breeding condition in the farm animals. Lower level of LH and selenium are equally modulators of repeat breeding case in farm animal. The relationship between the changes in hormonal profile and mineral profile of plasma support the alterations in ovarian function which may be the cause of repeat breeding in crossbred cows.

## Authors’ Contributions

AB carried out research work under the guidance of SB. SG helped in collection of sample and performed statistical analysis. AB drafted and revised the manuscript under the guidance of DS and SC. All authors read and approved the final manuscript.
